# Clinical outcomes in outpatient respiratory syncytial virus infection in immunocompromised children

**DOI:** 10.1111/irv.12375

**Published:** 2016-03-23

**Authors:** Helen Y. Chu, Jennifer Chin, Jessica Pollard, Danielle M. Zerr, Janet A. Englund

**Affiliations:** ^1^ Division of Allergy & Infectious DiseasesUniversity of WashingtonSeattleWAUSA; ^2^ Division of Infectious DiseasesSeattle Children's HospitalSeattleWAUSA; ^3^ Pediatric Hematology/OncologySeattle Children's HospitalSeattleWAUSA; ^4^ Present address: Maine Children's Cancer ProgramScarboroughMaineMEUSA

**Keywords:** Hematopoietic stem cell transplant, immunocompromised, outpatient, pediatric, respiratory syncytial virus

## Abstract

**Background:**

Immunocompromised patients are at high risk for morbidity and mortality due to respiratory syncytial virus (RSV) infection. Increasingly, pediatric patients with malignancy or undergoing transplantation are managed primarily as outpatients. Data regarding the clinical presentation and outcomes of RSV in the outpatient pediatric immunocompromised population are limited.

**Methods:**

We performed a retrospective cohort study of children with hematologic malignancy or hematopoietic or solid organ transplant with laboratory‐confirmed RSV infection diagnosed as outpatients at an academic medical center between 2008 and 2013.

**Results:**

Of 54 patients with RSV detected while outpatients, 15 (28%) were hospitalized, 7 (13%) received ribavirin, and one (2%) received intravenous immunoglobulin. One (2%) patient was critically ill, but there were no deaths due to RSV infection. Fever (*P* < 0·01) was associated with increased risk of hospitalization.

**Conclusions:**

Most immunocompromised children with RSV detected while outpatients did not require hospitalization or receive antiviral treatment. Potential studies of RSV therapies should consider inclusion of patients in an ambulatory setting.

## Introduction

Respiratory syncytial virus (RSV) is the most common cause of lower respiratory tract infection in US children under 1 year of age.^1^, ^2^, ^3^ Increased morbidity and mortality have been reported in high‐risk patients, such as premature infants, infants with cardiac disease, and severely immunocompromised patients.^4^, ^5^, ^6^ Current therapeutic options for the treatment of RSV are limited to ribavirin and/or intravenous immunoglobulin (IVIG).^7^, ^8^ New antivirals directed against RSV are under development with efficacy demonstrated in several human challenge studies in adults.^9^, ^10^ Increasingly, pediatric patients with malignancy or those undergoing transplantation are managed in the outpatient cancer care setting. Characteristics and clinical outcomes of RSV infection in pediatric immunocompromised outpatients may be different from acutely ill hospitalized inpatients. The objective of our study was to describe the clinical presentation and outcomes of RSV infection in an immunocompromised outpatient pediatric population.

## Methods

Using laboratory records, we identified patients between birth and 21 years of age who had laboratory confirmation of RSV by direct fluorescent antibody (DFA), real‐time reverse transcriptase‐polymerase chain reaction (qRT‐PCR), or viral culture at Seattle Children's Hospital in Seattle, WA, USA, between 2008 and 2013. For RSV quantitative viral load, the PCR threshold cycles of the nasal swab samples were compared to those of a standard curve generated by amplification of known numbers of RNA transcripts of the PCR amplicons.^11^, ^12^

We included patients with hematologic malignancy, solid organ transplant (SOT), or hematopoietic cell transplant (HCT) who were outpatient at the time of diagnosis. Sociodemographic, clinical, laboratory, and radiologic data were abstracted from the electronic medical chart using a standardized form in Project REDCap.^13^ An illness episode was defined as the presence of at least one respiratory symptom (cough, wheezing, increased work of breathing, rhinorrhea, and/or apnea) in a patient with RSV detected by laboratory testing. The end of the illness episode was defined as a minimum of 14 days following symptom resolution. Only the first RSV illness episode for each patient was included in this analysis. RSV‐associated hospitalization was classified based on provider documentation of reason for hospitalization in the medical record.

Potential healthcare‐associated infection was defined as an RSV illness in a patient seen in clinic two to eight days prior to RSV detection (“potential clinic acquired”) [18]. Neutropenia was defined as an absolute neutrophil count (ANC) < 500 cells/μl. Lymphopenia was defined as an absolute lymphocyte count (ALC) < 500 cells/μl. Viral or bacterial coinfections were determined by chart review of laboratory results obtained within 48 hours of diagnosis. Chest imaging obtained within seven days of diagnosis was included. Abnormal chest imaging was defined as a radiology result of consolidation, alveolar infiltrates, or airspace opacities. RSV‐attributable mortality was defined as death due to RSV‐associated respiratory failure. Data were analyzed using stata 12·1 (STATA Corp, College Station, TX, USA). Fisher's exact tests were used for comparison of categorical variables, and Wilcoxon rank sum and anova tests were used for comparison of continuous variables. This study was approved by the Institutional Review Board of Seattle Children's Hospital.

## Results

**Figure 1:**
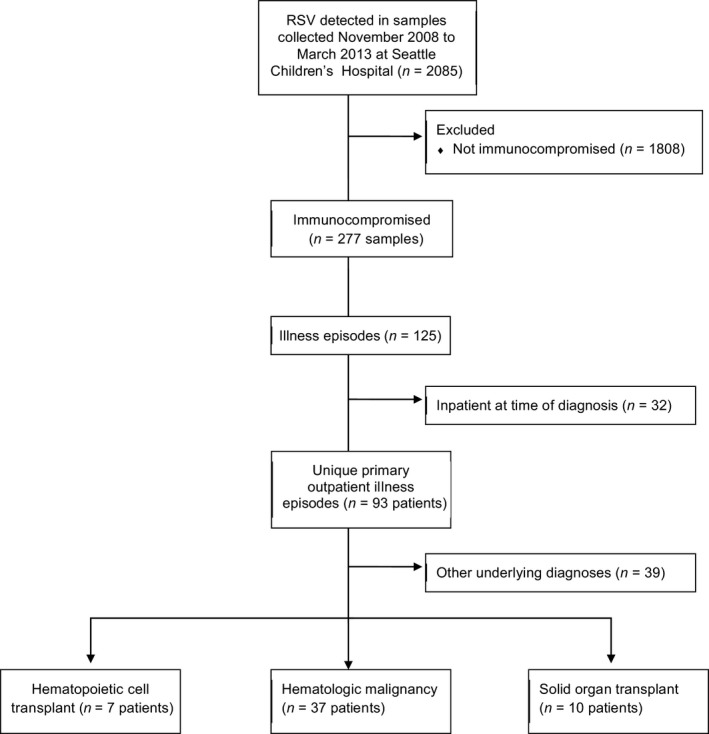
Flow diagram of study.

A total of 2085 respiratory samples with RSV detected were collected from children from birth to 21 years of age at Seattle Children's Hospital–University of Washington from November 2008 to March 2013 (Figure 1). Of these, 277 samples were collected from 125 immunocompromised patients, of whom 32 were inpatients at time of diagnosis and 39 had an immunocompromising condition other than a hematologic malignancy or transplant. A total of 67 samples were collected from 54 immunocompromised outpatients. Thirty‐seven (69%) patients had a hematologic malignancy, 10 (19%) were SOT recipients, and 7 (13%) were HCT recipients (Table 1). The majority of patients with a hematologic malignancy were actively receiving chemotherapy (*n* = 31; 84%). The median age of these patients was 6 years (range, 10 months–21 years). Of the 15 (28%) outpatients who were hospitalized due to RSV infection, six had a hematologic malignancy, three were SOT recipients, and six were HCT recipients. Thirty‐seven (69%) of RSV cases were potentially clinic acquired; the majority of these were in patients with a hematologic malignancy (*n* = 27; 73%). Patients with a fever were more likely to be hospitalized [13 (87%) vs. 14 (36%), respectively; *P* < 0·01], while those with a hematologic malignancy were less likely to be hospitalized [6 (40%) versus 31 (79%); *P* < 0·01; Table 2]. No patients were receiving palivizumab for RSV prophylaxis at the time of diagnosis. One (2%) patient with an underlying diagnosis of B‐cell ALL received IVIG 33 days prior to her RSV diagnosis.

**Table 1:** Characteristics of immunocompromised patients with respiratory syncytial virus (RSV) infection acquired as outpatients, stratified by underlying disease

Characteristic	Hematologic malignancy n=37 (69%); median (range) or n(%)	SOT n=10 (19%); median (range) or n(%)	HCT n=7 (13%); median (range) or n(%)
Median age in years	7·8 (2·5–21·3)	4·8 (1·0–14·9)	6·0 (0·8–9·1)
Female sex	19 (51)	5 (50)	2 (29)
Acquisition^a^			
Community acquired	8 (57)	3 (21)	3 (21)
Clinic acquired	27 (73)	7 (19)	3 (8)
Hospitalized due to RSV	6 (40)	3 (20)	6 (40)
Median length of hospital stay (range)	3·5 (2–6)	3 (3–3)	5 (1–11)
Active disease at diagnosis	31 (84)	8 (80)	7 (100)
Symptoms at presentation			
Fever	16 (43)	6 (60)	5 (71)
Cough	27 (73)	7 (70)	7 (100)
Rhinorrhea	24 (65)	4 (40)	6 (86)
Wheezing	2 (5)	1 (10)	2 (29)
Increased work of breathing	1 (3)	2 (20)	0 (0)
Receipt of oxygen	0 (0)	1 (33)	2 (67)
Neutropenia^b^	6 (17)	0 (0)	0 (0)
Lymphopenia^b^	6 (19)	0 (0)	0 (0)
RSV Detection Method			
DFA	31 (84)	9 (90)	5 (71)
PCR	4 (11)	1 (10)	3 (43)
Culture	21 (57)	3 (30)	2 (29)
RSV viral load in log_10_ copies/ml (range) (*n* = 7)	7·1 (5·9–7·6)	5·3 (5·3–5·3)	5·0 (4·9–8·3)
Abnormal chest imaging	4 (40)	5 (50)	1 (10)
Coinfection^c^	4 (40)	1 (10)	5 (50)
Receipt of ribavirin	3 (43)	0 (0)	4 (57)
Receipt of IVIG	1 (100)	0 (0)	0 (0)
Receipt of antibiotics	8 (22)	2 (20)	1 (14)
ICU admission	1 (3)	0 (0)	0 (0)

HCT, hematopoietic cell transplant recipient; SOT, solid organ transplant recipient; DFA, direct fluorescent antigen; PCR, polymerase chain reaction; IVIG, Intravenous immunoglobulin; ICU, intensive care unit stay.

a Community acquired infection defined as RSV detected less than 2 days after a clinic visit or more than 8 days after a clinic visit. Clinic acquired infection defined as RSV detected 2–8 days after a clinic visit.

b Neutropenia and lymphopenia defined as ANC < 500 and ALC < 500, respectively.

c Other coinfections were adenovirus (*n* = 4), rhinovirus (*n* = 3), parainfluenza 1–4 (*n* = 1), coronavirus (*n* = 1), and coagulase‐negative staphylococcus (*n* = 1).

**Table 2:** Comparison of characteristics of outpatients who did or did not require hospitalization for RSV illness

Variable	No hospitalization *n* = 39 median (range) or n (%)	Hospitalized due to RSV *n* = 15 median (range) or n (%)	P‐value
Median age in years	7·5 (1·0–21)	6·0 (0·8–18)	0·13
Hematologic malignancy	31 (79)	6 (40)	<0·01
Fever	14 (36)	13 (87)	<0·01
Lymphopenia	5 (11)	1 (2)	0·66
Neutropenia	3 (6)	3 (6)	0·33
Abnormal chest imaging	6 (60)	4 (40)	0·70

### Management of patients with RSV

Ten (27%) of 37 patients with chest imaging performed had abnormalities noted, with alveolar infiltrates (*n* = 4; 40%) as the most common finding. Three (6%) patients required supplemental oxygen therapy, and no patients required mechanical ventilation. Eleven (20%) patients received antibiotic therapy; indications included febrile neutropenia, bacteremia, and pneumonia.

A minority of patients (*n* = 8, 15%) received ribavirin and/or IVIG for a median of 4·5 days (range, 3–31 days) starting 1 day (range, 0–4 days) after RSV detection. Indications for treatment with ribavirin or IVIG included lymphopenia, delayed intensification phase of chemotherapy, neutropenia, hypogammaglobulinemia, and preparation for transplant. One patient (2%) was admitted to the ICU, and no patients died due to RSV infection; however, one (2%) patient infected with RSV died due to complications of her underlying condition 676 days after RSV diagnosis.

### RSV viral load over time

**Figure 2:**
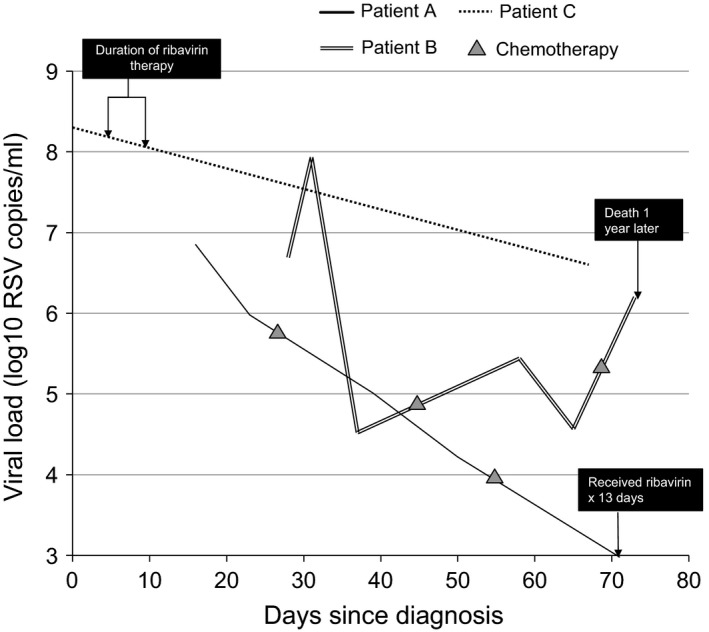
Viral load over time in three patients with repeated sampling during their RSV illness episode.

Seven patients had testing performed for RSV quantitative viral load. The median initial RSV viral load was 5·9 log_10_ copies/ml (range, 4·9–8·3). Of the 3 patients with sequential viral load testing (Figure 2), Patient A, an 11‐year‐old female HCT recipient, received a 13‐day course of aerosolized ribavirin 2 days after which a RSV viral load was measured at 3·87 log_10_ RSV copies/ml. Patient B, a 10‐year‐old female with ALL, had an increase in RSV viral load from 4·52 log_10_ RSV copies/ml to 5·44 log_10_ RSV copies/ml after an initial course of chemotherapy. After her second round of chemotherapy, her RSV viral load increased from 4·57 log_10_ RSV copies/ml to 6·24 log_10_ RSV copies/ml. Patient C, a 3‐year‐old male HCT recipient, experienced a decrease in viral load from 8·34 log_10_ RSV copies/ml to 6·55 log_10_ RSV copies/ml after a 6‐day course of ribavirin.

## Discussion

The vast majority of immunocompromised pediatric patients with RSV diagnosed at a tertiary care hospital over a 5‐year period were outpatients who were followed and diagnosed in outpatient specialty clinics and were not hospitalized following the detection of RSV. In contrast to reports of RSV infection in hospitalized immunocompromised children, these patients were less likely to be severely immunocompromised given their outpatient status. Few patients in our study received ribavirin and/or IVIG or supportive care with supplemental oxygen, although evaluation of these patients included chest imaging in 37% and antibiotic use in 20%. The overall impact of RSV alone on receipt of chemotherapy or immunosuppressive therapy was difficult to assess, but in this group of immunocompromised middle‐aged children who were likely to have previously experienced RSV infection, the immediate consequences of RSV infection were generally not severe.

No child died in our study due to RSV‐related causes in our study. Previous studies reported mortality rates ranging from 3 to 5% in patients with chronic lung disease or congenital heart disease to 12–55% in HCT recipients.^14^, ^15^ In a single‐center study of pediatric immunocompromised inpatients, 5% mortality was reported, with an ICU admission rate of 28%.^16^ We believe that the low mortality in our population is due to the outpatient population, as well as advances in supportive care and more sensitive laboratory diagnostic techniques that enable more timely and sensitive detection of RSV infection.^12^

Fever was associated with risk of hospitalization in our study, while patients with hematologic malignancy were less likely to be hospitalized. El‐Saleeby and colleagues found that lymphopenia and younger age were risk factors for RSV‐associated LRTI, neither of which was associated with hospitalization in our study.^17^ Compared to the El‐Saleeby study, we also had a higher proportion of SOT recipients, who may be less immunocompromised depending on time since transplant and degree of immunosuppression. In this study, only a minority of the solid organ transplant recipients in this study were hospitalized and none died. Single institution inpatient studies have reported that RSV is the most commonly detected respiratory virus, particularly in pediatric lung transplant recipients. Pediatric abdominal transplant recipients with RSV infections have been reported to have mortality rates of 40%^18^, ^19^, ^20^; however, these studies have primarily described hospitalized patients. Larger studies in solid organ transplant recipients are needed to address this issue more completely.

Because this was an observational study, we were unable to determine the treatment efficacy of ribavirin or IVIG on viral load. We did observe that viral load decreased after initiation of ribavirin treatment in one patient and increased after several rounds of chemotherapy in one patient. RSV in immunocompromised patients is associated with prolonged shedding, likely as a consequence of inability of the host immune response to clear the infection.^21^ To our knowledge, there are no large‐scale retrospective or prospective studies of antiviral therapy for RSV in immunocompromised pediatric populations. With several new RSV antivirals in development including two that have been evaluated in challenge studies in healthy young adults, the assessment of sequential viral loads with receipt of treatment should be assessed to correlate with clinical outcomes in this relatively high‐risk patient population.

We found high rates of RSV infection that could have potentially been acquired in the outpatient clinic setting. RSV is often implicated in inpatient and outpatient nosocomial outbreaks in transplant wards, as documented by genotypic analysis.^22^, ^23^ Although these cases in our study could have been linked to clinic visits, these children were also living at home and therefore likely to have had more frequent contact with individuals interfacing with the community, such as siblings. In addition, many patients were seen very frequently, at least weekly, per their treatment protocols, which could also explain the high rate of potentially clinic‐acquired RSV infection.

Limitations of our study include the use of retrospective chart review at a single institution. It is possible that patients who did not present for care with RSV infections were omitted from the study; however, most immunocompromised children received all of their care at our institution. It is also possible that we missed cases of RSV due to the limited sensitivity of DFA as compared to RT‐PCR assay in earlier years of the study. However, at our institution, DFA has a sensitivity of 93% compared to RT‐PCR in young children.^11^ Additional limitations include an inability to discern true nosocomial clinic‐acquired infections; sequencing of the samples would need to be performed to identify identical viral strains, and no residual samples were available for this analysis.

In conclusion, we find that a significant burden of RSV disease occurs in outpatient immunocompromised children, with low hospitalization rates and no mortality in a 5‐year study at our center. Studies of therapeutic interventions, such as trials of new antivirals, should consider inclusion of both outpatient and inpatient immunocompromised populations to assess the impact of treatment on a broadly representative patient population.

## Conflict of interests

HYC has received funding for research unrelated to this work from Pfizer and Glaxo Smith Kline; DMZ has received funding for research unrelated to this work from Chimerix; JAE has received reimbursement for speaking by Abbvie, and has acted as a paid consultant for Gilead and Pfizer, Inc. JAE's institution has received research support from Gilead, Pfizer, Inc., and GlaxoSmithKline unrelated to this work.

## Funding

This study was supported by a grant from the National Institutes of Health, USA (K23AI103105 to HYC) and UL1TR000423 (Project RedCap).
